# Betaglycan (TβRIII) Is Expressed in the Thymus and Regulates T Cell Development by Protecting Thymocytes from Apoptosis

**DOI:** 10.1371/journal.pone.0044217

**Published:** 2012-08-29

**Authors:** German R. Aleman-Muench, Valentin Mendoza, Kaye Stenvers, Eduardo A. Garcia-Zepeda, Fernando Lopez-Casillas, Chander Raman, Gloria Soldevila

**Affiliations:** 1 Departamento de Inmunología, Instituto de Investigaciones Biomédicas, Universidad Nacional Autónoma de México, México, México; 2 Departamento de Biología Celular, Instituto de Fisiología Celular, Universidad Nacional Autónoma de México, México, México; 3 Reproductive Development and Cancer laboratory, Prince Henry′s Institute of Medical Research, Clayton, Victoria, Australia; 4 Departments of Medicine and Microbiology, Division of Clinical Immunology and Rheumatology University of Alabama at Birmingham, Alabama, United States of America; University Paris Sud, France

## Abstract

TGF-β type III receptor (TβRIII) is a coreceptor for TGFβ family members required for high-affinity binding of these ligands to their receptors, potentiating their cellular functions. TGF-β [1]–[3], bone morphogenetic proteins (BMP2/4) and inhibins regulate different checkpoints during T cell differentiation. Although TβRIII is expressed on hematopoietic cells, the role of this receptor in the immune system remains elusive. Here, we provide the first evidence that TβRIII is developmentally expressed during T cell ontogeny, and plays a crucial role in thymocyte differentiation. Blocking of endogenous TβRIII in fetal thymic organ cultures led to a delay in DN-DP transition. In addition, *in vitro* development of TβRIII^−/−^ thymic lobes also showed a significant reduction in absolute thymocyte numbers, which correlated with increased thymocyte apoptosis, resembling the phenotype reported in Inhibin α ^−/−^ thymic lobes. These data suggest that Inhibins and TβRIII may function as a molecular pair regulating T cell development.

## Introduction

T cell development requires the recognition of self-peptide MHC complexes by immature thymocytes, leading to the selection of a self-restricted and autotolerant T cell repertoire. In addition to the nature of TCR signals triggered by self-peptide recognition, other signals provided by thymic stromal cells, such as those triggered by members of TGF-β superfamily like TGFβ, activins/inhibin and BMP subfamilies have been shown to act as key regulators of apoptosis, survival and cell cycle progression in different cell types [1]–[3].

We and others have described that members of TGF-β superfamily are differentially expressed in the thymus and regulate specific developmental checkpoints, influencing T cell development in [4], [5]. Specifically, among TGFβs, only TGFβ1 and TGFβ2 appear to regulate DN1-DN2 and DN-DP transitions and promote maturation of CD8SP [4]. On the other hand, BMPs and their negative regulators, chordin, noggin and twisted gastrulation (Tsg), are also expressed in the thymus. BMP2 and BMP4 were shown to negatively regulate DN1-DN2, DN3-DN4 and DN-DP transitions [4], [5]. Finally, we have recently described that inhibins are abundantly expressed in the thymus by stromal cells and thymocytes [6] and that, addition of exogenous inhibins in FTOCs regulate T cell development at the DN3-DN4, DN-DP, and DP-CD8SP stages [7]. Moreover, endogenous inhibins were required to obtain normal thymocyte numbers and adequate DN-DP transition during *in vitro* T cell development [7].

A central coreceptor in the canonical signaling pathway of TGF-β is betaglycan, also known as the TGF-β type III receptor (TβRIII), which is a widely expressed membrane-anchored proteoglycan. Structurally, it is characterized by a large extracellular region, containing heparan and chondroitin sulphate chains, and a short cytoplasmic domain that lacks a signaling motif, which has recently been shown to regulate cell processes like apoptosis and cell migration [8], [9]. TβRIII-null mice embryos die between E13.5 to E18.5 of embryonic stage by heart and liver defects, caused by an altered TGF-β2-induced mesenchymal transformation process and the incidence of apoptotic events [10]. Recently, it has been described that the absence of TβRIII also compromises normal seminiferous cord formation, Leydig cell function in testis [11] and alters kidney development [12].

The main function of TβRIII is to orchestrate the TGFβ, BMP and inhibin-mediated signals in different cell types. TβRIII enhances the binding of all three TGF-β isoforms to the TGF-β signaling receptor complex, but is specially required for high affinity binding and functional activity of TGF-β2 [10], [13]–[17]. On the other hand, TβRIII enhances the binding of BMP2, BMP4, BMP7 and GDF5 to BMPR1 leading to an increase in Smad1 phosphorylation, and thus potentiates its functional effects [18]. In addition, TβRIII binds inhibins with high affinity, potentiating the exclusion of ACTRIB (ALK4) to antagonize activin-mediated functions [14], [19]–[21]. Also, TβRIII enables inhibins to antagonize BMP signaling [22] and to reduce TGF-β2 signals through the endocytic internalization of TβRIII [23]. Conversely, TβRIII also allow TGF-β1 and 2 to attenuate inhibin-mediated functions by downregulating the expression and binding of this co-receptor [24], [25].

Although TβRIII is broadly expressed in many tissues, its presence and the potential function of this receptor in the hematopoietic system remains poorly characterized [26]–[31]. Given that downstream signaling of many TGF-β ligands are regulated by TβRIII to fine tune key cellular processes, here we investigated the expression of TβRIII in the thymus and its potential role in T cell differentiation.

**Figure 1:**
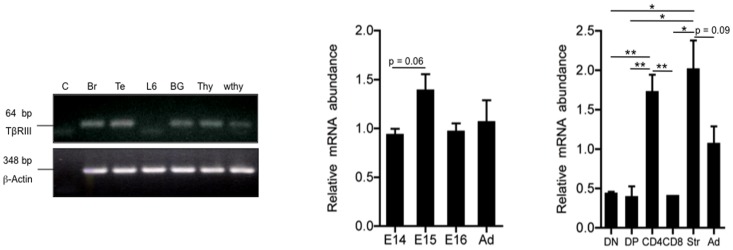
TβRIII mRNA is expressed in fetal and adult murine thymus. RT-PCR was employed to amplify a TβRIII product (64 bp) from cDNA obtained of control and test samples; as positive control, Brain (Br), Testis (Te), TβRIII stably transfected myoblast line cell (BG) were used. H_2_O (c) and the parental, TβRIII-negative, myoblast cell line L6E9 (L6) were used as negative controls. cDNA of total thymocytes (thy) and whole adult thymus (wthy, including thymocytes and stromal cells) were tested for the expression of TβRIII. Quantitation of TβRIII expression was performed by quantitative real time PCR analysis of cDNA samples obtained from E14, E15 and E16 thymi and whole adult thymus. Real time PCR shows differential TβRIII expression in sorted thymocyte subpopulations DN (CD4^−^CD8^−^), DP (CD4^+^CD8^+^), CD8^+^ SP and CD4^+^ SP. Str (thymic stroma), Ad (adult thymus). The purity of all sorted thymocyte subsets was greater than 96%. Values represent relative expression (2^−Δct^), expressed as mean values ± SEM of three independent experiments. Asterisks indicate * p≤0.05, and **p≤0.01.

## Materials and Methods

### Mice

4 to 6 week old C57BL/6 mice were used in our experiments. TβRIII wild type, heterozygous, and null mouse embryos [10] were obtained from synchronized embryonic day 14 (E14) matings of TβRIII heterozygous mice. All animal handling and experimental procedures were done according to the Instituto de Investigaciones Biomedicas ethics guidelines. The study was approved by the “Comité para el Cuidado y Uso de Animales de Laboratorio (CCUAL)” of the Institute.

**Figure 2:**
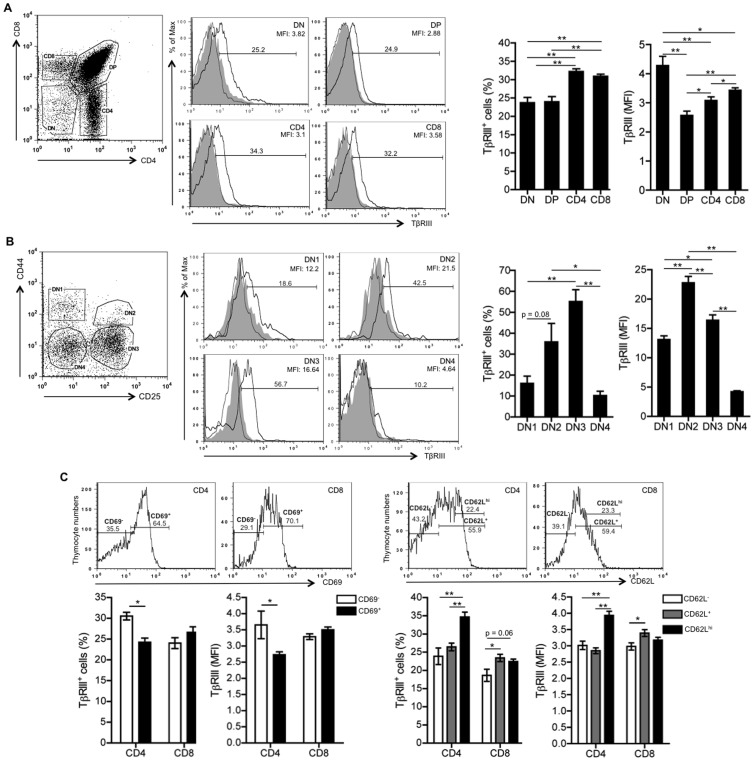
TβRIII is developmentally expressed during T cell ontogeny. Thymocytes from 4 to 6 week old C57BL/6 background mice were stained with antibodies to CD4, CD8, and TβRIII. Pre-immune serum was used as an internal background staining control. (A) Representative histograms showing the percentage of TβRIII^+^ cells in gated DN, DP, CD4^+^ SP and CD8^+^ SP subsets. Graphs show the percentage of TβRIII^+^ thymocytes and geometric MFI calculated after subtracting the background staining. (B) Representative histograms showing the percentage of TβRIII^+^ thymocytes in DN1, DN2, DN3 and DN4 immature subsets. Graph represents the analysis of TβRIII^+^ cells and geometric MFI in gated DN1, DN2, DN3 and DN4 immature subsets. Unstained (filled curve in gray), preimmune serum (gray line) and anti-TβRIII antiserum (black line). Data are representative of 4 independent experiments. (C) Left panel, graph shows the percentage of TβRIII^+^ cells and geometric MFI in gated CD69^−^ and CD69^+^ SP thymocytes as showed in histograms. Right panel, graph represents the analysis of TβRIII^+^ cells and geometric MFI in gated CD62L^−^, CD62L^+^ and CD62L^hi^ SP thymocytes as showed in upper panel. Mean values ± SEM are shown (n  = 5 per group). Asterisks indicate * p≤0.05, and **p≤0.01.

### PCR and Genotyping

Amplification of wild-type or mutant TβRIII alleles in tissue samples from E14 embryos was achieved with the following primers: P1 5′-ATTGTGTTCATAGGTCCAGA-3′, wt 5-CCTAGTCCTTGGTCTGTACT-3′, and Neo 5′- TAGGGTTCCGATTTAGTGCT- 3′, using the following program: 1 minute of initial denaturing step at 94°C, then 35 cycles of denaturing (94°C for 1 min), annealing (53°C for 1 min), and elongation (72°C 1 min), and one final step at 72°C for 5 min. Products obtained after PCR were electrophoresed in 1% agarose gels.

**Figure 3:**
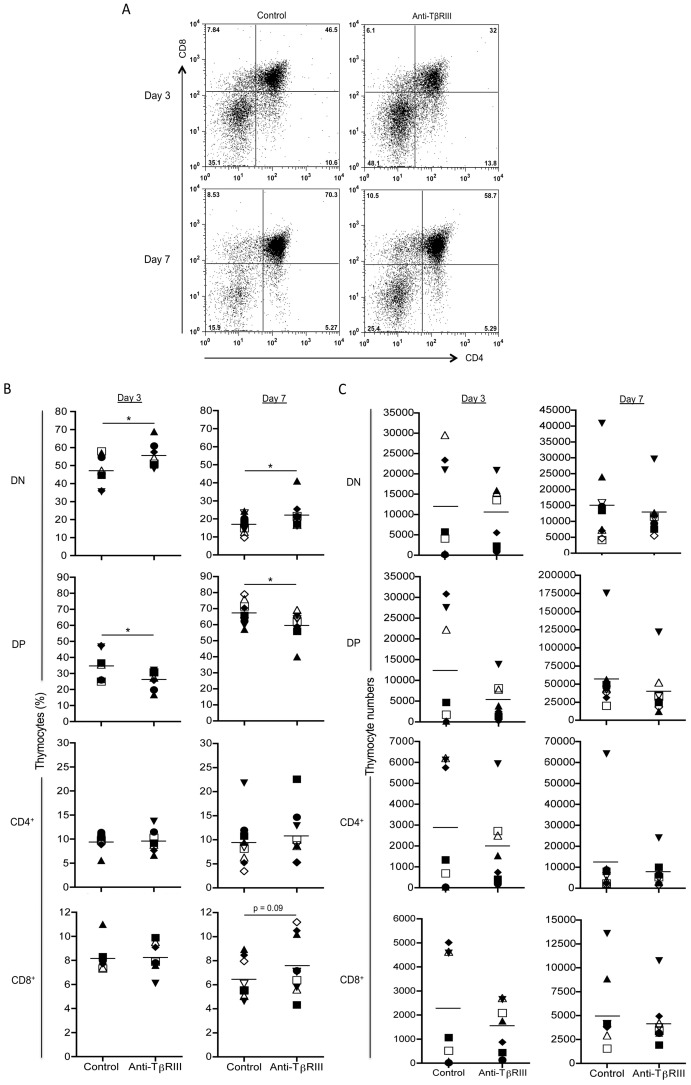
The blocking of TβRIII in FTOCs alters T cell development. E14 thymic lobes were cultured in the presence of anti-TβRIII antibody or in the presence of pre-immune serum (control lobe). At day 3 and 7 of culture thymic lobes were disaggregated, counted and stained with antibodies to CD4, CD8. (A) Representative CD4 versus CD8 staining dot plots. (B). Comparative graphs represent the percentages of DN, DP, CD4SP and CD8SP thymocytes obtained after 3 and 7 days of culture between both treatments. (C) Analysis of cell numbers in non-treated and anti-TβRIII treated FTOCs at day 3 and 7. Data are representative of two independent experiments. Mean values ± SEM are shown (n  = 7 per group for day 3, and n  = 9 per group for day 7). Asterisks indicate *p≤0.05.

### RNA Isolation and RT-PCR

Total RNA was obtained from total fetal (E14-E15 gestation) and adult thymi, E15 thymic stromal cells, sorted adult thymocyte subpopulations, L6E9 and BG22 myoblast cell lines. Testis and brain were used as control tissues. E15 thymic stromal cells were purified as previously described [6]. Sorted thymocyte subsets (DN, DP, CD4^+^ and CD8^+^) were obtained with FACS Aria cell sorter (BD Biosciences) with a purity of >95%. RNA was isolated using RNA-STAT60 reagent (Tel-Test Inc, Friendslaw, TX) according to manufacturer’s protocol. 5–10 µg of total RNA was treated with “DNA-Free” reagent (Ambion Inc. Austin TX). cDNA was synthetized using M-MLV RT and oligo dT (both from Invitrogen Inc, Carlsbad, CA) according to manufacturer’s recommendations.

### Real Time PCR Analysis

The following primers were used: TβRIII Forward 5′ -GCCAGACGGCTACGAAGATTT- 3′, TβRIII reverse 5′-AACACTACCACTCCAGCACGG- 3′, β-Actin Forward 5′ -TGGAATCCTGTGGCATCCATGAAAC- 3′ and β-Actin Reverse 5′- TAAAACGCAGCTCAGTAACAGTCCG- 3′. Measurement of gene expression was performed amplifying cDNA with SYBR Green PCR Core Kit (Applied Biosystems) and analyzed with the ABI PRISM 7000 Sequence Detection System software (Applied Biosystems, Foster City, CA). Amplification conditions for TβRIII and β-actin were an initial step of 5 min at 95°C, followed by 40 cycles of 95°C for 30 sec, 58°C for 1 min and 61.3°C for 1 min. Expression specific gene was calculated using the formula 2^−(ΔCt)^. The calculation of TβRIII expression was normalized to β-actin and performed as previously described [6]. The PCR products were analyzed on a 2% (w/v) agarose gel to confirm purity and size of products.

### Flow Cytometry

A total of 0.5-1×10^6^ cells were treated with Fc block for 30 minutes at 4°C following by staining with the indicated antibodies: FITC-coupled anti-CD4 (RM4-5), PE-coupled anti-CD44 (Pgp-1, Ly-24), PE-coupled anti-CD4 (L3T4), PE-coupled anti-CD8 (Ly-2), PE-coupled anti-CD19 (1D3), biotin-conjugated anti-CD25 (7D4), PeCy5-coupled anti-CD4 (L3T4), Cy5-coupled anti-CD8 (Ly-2), APC-coupled anti-CD4 (RM4-5) and PE-conjugated rabbit anti-active caspase 3, all from BD Biosciences (San Jose, CA) obtained from BD Biosciences and Biolegend. For TβRIII detection a polyclonal antibody was used [13]. As secondary reagents, FITC-conjugated goat anti-rabbit IgG (Invitrogen), PE-conjugated anti-rabbit IgG (Invitrogen) and APC-conjugated streptavidin (BD Biosciences) were used. Dead cells were gated out depending on forward scattering (FSC) and side scattering (SSC). All samples were captured in a FACsCalibur and FACSAria (BD) and data were analyzed with FlowJo© Tree Star software.

### Western Blotting Analysis

Cell lysates (10×10^6^ cells) were resolved on a 10% sodium dodecyl sulphate–polyacrylamide gel electrophoresis (SDS–PAGE) and transferred to Immobilon-P membranes (Millipore, Billerica, MA). Immunoblotting was carried out with anti-TβRIII (Cell Signaling Tech Inc., Beverly, MA), followed by horseradish peroxidase-labeled anti-mouse immunoglobulin (Amersham, Buckinhamshire, UK). As a loading control, anti-β-actin antibody was used (kindly provided by Dr. Isaura Meza, CINVESTAV-IPN, D.F, México). Immunoblots were revealed by enhanced chemiluminescence assay (ECL, Amersham).

### FTOCs

Fetal thymi of TβRIII wild type, heterozygous, and null mouse embryos were obtained from timed matings of heterozygote mice (C57BL/6 background) and genotyped as described above. Fetal thymi were cultured as previously described [7]. Fetal thymi were cultured with or without anti-TβRIII blocking Ab (1/100 dilution) for 3–7 days, and medium was refreshed every third day. On days 0, 3 and 7 of culture, thymic lobes were disaggregated and thymocytes counted and stained for flow cytometric analysis.

### Apoptosis Assays

Thymocytes were initially stained with CD4/CD8 antibodies followed by Annexin-V, according to the manufacturer’s protocol (BD Biosciences). For the analysis of active caspase 3, cells were permeabilized with Fixation/Permeabilization solution (BD Biosciences) for 1 hr at 4°C and then incubated with anti-active caspase 3-PE (eBioscience, San Diego, CA) for 30 minutes at 4°C.

### Statistical Analysis

For FTOCs using the antibody anti-TβRIII, a paired two-tailed Student’s test was used to compare lobes from each fetus (control lobe versus treated lobe). For the rest of experiments, an unpaired Student`s test was used. Asterisks indicate p≤0.05(*) and p≤0.01(**). P values ≤0.05 were considered as statistically significant.

**Figure 4:**
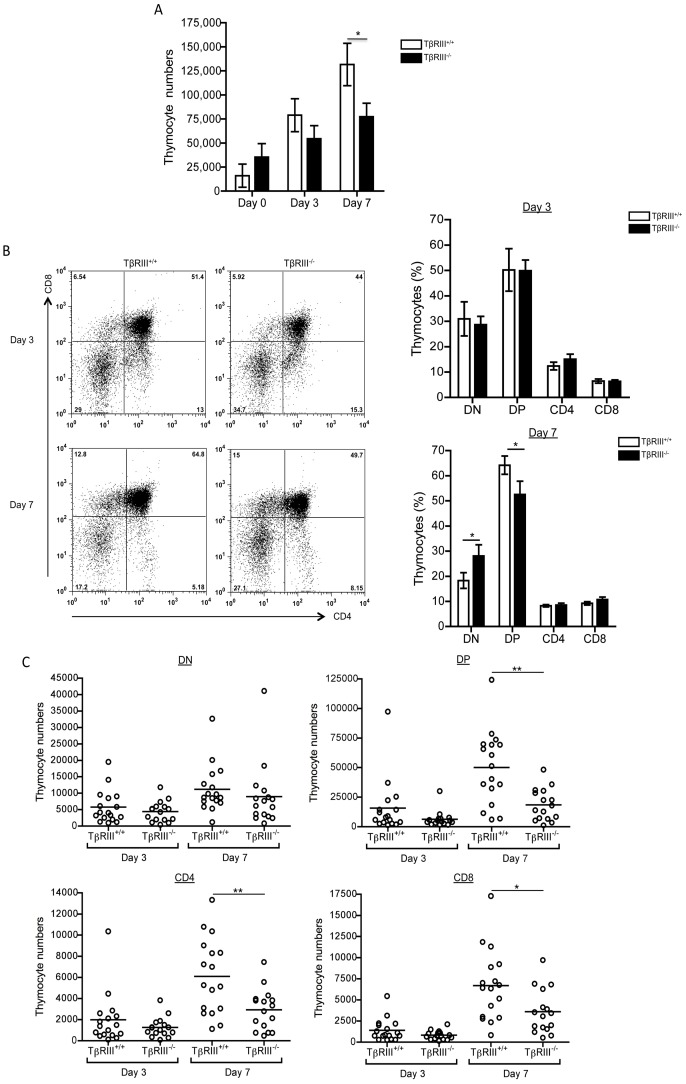
TβRIII^−/−^ fetal thymi display an altered T cell development. (A) Graph of thymocyte numbers at days 0, 3 and 7 from E14 TβRIII^+/+^ and TβRIII^−/−^ fetal thymic lobes, respectively. (B) Representative CD4 versus CD8 staining dot plots from TβRIII^+/+^ and TβRIII^−/−^ fetal thymic lobes at days 3 and 7 of culture. Summary of experiments showing the percentage of each thymocyte subsets from TβRIII^+/+^ and TβRIII^−/−^ FTOCs at days 3 and 7. (C) Analysis of total cell numbers in gated DN, DP and CD4SP and CD8SP thymocytes from TβRIII^+/+^ and TβRIII^−/−^ fetal thymi. Data are representative of three independent experiments. Mean values ± SEM are shown, TβRIII^+/+^ n  = 17 and TβRIII^−/−^ n  = 16. Asterisks indicate *p≤0.05 and **p≤0.01.

## Results and Discussion

### TβRIII mRNA is Expressed in Murine Thymocytes and Stromal Cells

We first investigated the expression of TβRIII in the thymus at the mRNA level. Although we expected to find expression on stromal lineage cells (which include epithelial cells, macrophages and dendritic cells), surprisingly, we found significant levels of TβRIII in thymocytes (Figure 1). This expression was comparable to levels observed in the brain and testis, which were used as control tissues [32]. TGFβ superfamily members have an essential function in early T cell development that initiates with the arrival of lymphoid progenitors to the thymic anlage from 13.5 days of gestation [4], [5]. In order to determine if TβRIII signaling might have a role in fetal thymocyte development, we analyzed the gene expression of the receptor in E14, E15 and E16 fetal thymi and compared it to that in adult thymi. We observed that TβRIII was significantly expressed in all fetal stages at levels equivalent to adult thymus. Although the expression of TβRIII was slightly elevated E15 fetal thymi, it was not significantly different from other time-points during development (Figure 1). To directly determine the expression of TβRIII in thymocytes at different stages of development from that in stromal cells, we sorted DN, DP, SP CD4^+^ and SP CD8^+^ thymocyte subsets from adult thymi and E15 thymic stromal cells. As expected, we observed that TβRIII mRNA was highly expressed in stromal cells (Figure 1). Remarkably, thymocyte subpopulations at all stages of development expressed significant levels of this proteoglycan with the CD4^+^ SP subset expressing the highest levels (Figure 1). Western blot analysis revealed confirmed a 100 kDa protein that corresponds to the core protein of mouse TβRIII in total adult thymocytes, lymph node cells and splenocytes (Figure S1A).

**Figure 5:**
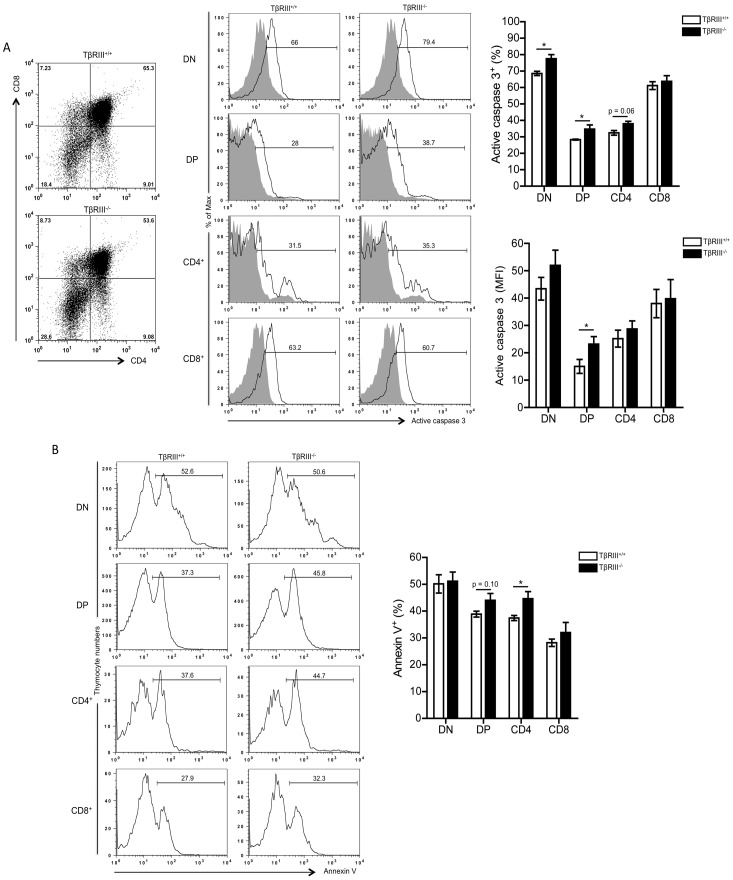
TβRIII deficiency results in increased apoptosis of developing thymocytes. (A) Left panel, representative CD4 versus CD8 staining dot plots from TβRIII^+/+^ and TβRIII^−/−^ fetal thymic lobes at day 7 of culture. Histograms show the expression of active caspase 3^+^ cells in each gated thymocyte subset. Right panel, graphs represent the percentage of active caspase 3^+^ cells and the levels of expression (MFI values) in each thymocyte subset. Data are representative of three independent experiments. (B) Left panel, representative histograms show the percentage of Annexin V^+^ cells in gated thymocyte subsets. Right panel, graph shows the analysis of the percentage of Annexin V^+^ cells in thymocytes from day 7 TβRIII^+/+^ or TβRIII^−/−^ FTOCs. Data are representative of two independent experiments. Mean values ± SEM are shown (TβRIII^+/+^ n = 3 and TβRIII^−/−^ n = 3). Asterisks indicate statistically significant differences (** p≤0.05).

Thus, this is the first evidence that ΤβRIII is expressed in thymic stromal cells and also in thymocytes, suggesting its potential role during T cell development.

### TβRIII Expression is Regulated during T Cell Ontogeny

Since type I and type II TGFβ receptors are differentially expressed in thymocyte subsets and their expression is associated with distinct thymocyte responsiveness to TGF-β superfamily ligands [4], [5], [33], we investigated whether TβRIII is differentially expressed on the cell surface of developing thymocytes. For this purpose we used a polyclonal antibody directed against the TβRIII ectodomain. Its specificity was confirmed by staining of TβRIII deficient fetal thymocytes and after competition with a soluble form of TβRIII (Figure S1B and C). Consistent with the mRNA expression data (Figure 1), all thymocyte subsets expressed TβRIII protein on the cell surface, showing an increased percentage of TβRIII^+^ cells within the SP subset (Figure 2A). Based on mean fluorescence intensity values (MFI), we observed that the DN subset expressed the highest levels of TβRIII, which diminished at DP stage and moderately increased at the SP stages (Figure 2A).

It is well known that TGFβs, activins/inhibins and BMPs regulate early stages of T cell differentiation [7], we therefore analyzed the expression of TβRIII in immature DN thymocytes by using anti-CD44 and anti-CD25 antibodies to identify DN1, DN2, DN3 and DN4 stages. As shown in Figure 2B, the percentage of DN cells expressing TβRIII cells increased from DN1 to DN2 reaching a peak at the DN3 stage followed by a significant decrease at the DN4 stage. Analysis of MFI confirmed the downregulation of TβRIII at DN4 stage, suggesting that pre-TCR signaling may regulate TβRIII expression in thymocytes. Consistent with these findings, we suggest that TβRIII expression at DN3 stage may be important to integrate the signaling output of BMPs and inhibins influencing the transition of thymocytes to the DP stage. In fact, we recently demonstrated that inhibin A, a ligand with high affinity for TβRIII, promoted DN3 to DN4 transition in FTOCs, suggesting their positive role during TCR β-selection process [7]. Moreover, an interesting mechanism was described for BMPs, where the expression of twisted gastrulation (Tsg) is induced after pre-TCR signaling to reverse the BMP2/4-dependent arrest and to promote T cell differentiation [34]. However, future experiments will be necessary to discriminate the contribution of TβRIII in each TGFβ ligand-mediated functions at this stage.

Since TβRIII is expressed in SP thymocytes, we next investigated whether upregulation of this receptor might accompany the process of positive selection. We used TCRβ, CD5 and CD69 as markers to analyze TβRIII expression in DP cells that have undergone positive selection. No correlation was observed between the expression of TβRIII and the levels of CD5, CD69 and TCRβ in DP thymocytes, suggesting that TβRIII expression in thymocytes is not modified by TCR signaling (data not shown). However, analysis of mature SP thymocytes indicated that TβRIII is upregulated in CD4^+^ SP CD69^−^ compared to CD4^+^ SP CD69^+^ thymocytes (Figure 2C, left panels), as well as in CD62L^hi^ CD4^+^ SP and CD62L^+^ CD8^+^ SP compared to CD62L^−^ cells (Figure 2C, right panels). Altogether, these data show that TβRIII expression may be associated with the terminal differentiation of thymocytes and is preferentially expressed in the most functionally mature CD4^+^ and CD8^+^ SP cells (CD69^−^ and CD62L^+/hi^, respectively).

### TβRIII Blocking Results in Delayed T Cell Development

TβRIII ligands TGF-βs, inhibins and some BMPs, have been reported to regulate specific checkpoints during T cell differentiation [4]. To directly determine if TβRIII has a role in T cell development, we evaluated the requirement of this co-receptor by performing blocking assays in FTOCs with specific antisera directed against its extracellular domain. This strategy has been previously employed to evaluate the requirement of Tβ RIII for epithelial-mesenchymal transition [17] and to restore the T cell stimulatory function of DCs suppressed by TGFβ [29]. No differences were observed in absolute cell numbers or in percentages of DN1-DN4 thymocytes at day 3 of culture under blocking conditions (Figure S2). In addition, when we examined T cell development at day 7 of culture, a significative decrease of DN2 subset and a slight reduction was observed in anti-TβRIII treated lobes (Figure S2C and D). Interestingly, blocking of TβRIII signaling significantly reduced the proportion of DP thymocytes that was associated with a corresponding increase in the DN subset (Figure 3A and B) indicating that TβRIII may act by regulating pre-TCR mediated signals, as has been reported for its ligands, BMPs and inhibins [7], [34]. This data correlate with the downregulation of TβRIII expression observed between DN3-DN4 and between DN and DP (Figure 2A and 2B). As shown in Figure 3C, analysis of thymocyte cell numbers showed a slight decrease, although not significant, in DP and CD4SP cell numbers at day 3 of culture in fetal lobes treated with anti-TβRIII antiserum.

### T Cell Development is Attenuated in TβRIII Null Embryos

To further confirm the role of TβRIII during thymocyte development, we performed FTOCs of TβRIII null embryos. TβRIII null mice show intrauterine lethality due to proliferative defects in heart and apoptosis in liver, beginning at E13.5 and with higher mortality by E16.5 [10]. Therefore to analyze the impact of TβRIII deficiency in thymocyte development and to reduce deleterious systemic defects, we isolated E14 fetal thymi obtained from TβRIII heterozygous pregnant females and analyzed T cell development in FTOC. We observed no differences in cell numbers at day 0 between all of the genotypes (Figure 4A). In addition, analysis of DN immature subsets showed no significant differences in the absence of TβRIII (Figure S3). However by day 3 and significantly by day 7, thymocyte numbers were greatly reduced in TβRIII null fetal thymi compared to wild type thymi. Consistently, analysis at day 7 of TβRIII^−/−^ FTOCs showed a significant reduction in the percentage of DP thymocytes, which was accompanied by an increase in DN thymocytes (Figure 4B), which correlates with the reduced cellularity observed in the absence of TβRIII (Figure 4A). In fact, analysis of cell numbers in TβRIII^−/−^ FTOCs at day 7 clearly show a significant decrease of DP and consequently in CD4SP and CD8SP thymocytes (Figure 4C). No differences in cell numbers between TβRIII^+/+^ and TβRIII^−/−^ fetal thymi were observed at day 3 of culture. (Figure 4C). This reduction in thymus cellularity is more pronounced in TβRIII^−/−^ FTOCs than under anti-TβRIII blocking conditions, suggesting that the antibody might not completely block all the available TβRIII (membrane bound and/or soluble) or alternatively, it may only affect membrane bound TβRIII-mediaded actions, while in TβRIII deficient thymocytes, also ligand-independent functions are absent, resulting in a more marked phenotype.

Although the kinetics are different, our results show that DN-DP transition is decreased when TβRIII signaling is compromised by use of blocking antibodies or by genetic deletion of the receptor. These data support the notion that TβRIII regulates DN-DP transition during thymocyte differentiation. In line with these findings, despite to the difficulty to discriminate the contribution of TβRIII in the actions mediated by each ligand during thymocyte development, we have recently demonstrated that inhibin α^−/−^ thymic lobes [7] also show reduced thymocyte numbers and a delayed DN-DP transition. We propose that TβRIII and inhibins may function as a molecular pair to regulate T cell differentiation, a functional association observed in other cell types [35].

### Increased Apoptosis in the Absence of TβRIII

Enhanced apoptosis of thymocytes in TβRIII^−/−^ thymic lobes might be the mechanism responsible for the impaired DN-DP transition and the reduced cellularity observed during thymocyte differentiation. This prediction arises from the study of fetal liver of TβRIII null embryos, which showed increased apoptosis associated with a significant reduction in cellularity in liver [10]. To directly test this, we analyzed the extent of ongoing apoptosis at day 7 of culture by measuring the levels of the active form of caspase 3 in all thymocyte subpopulations (Figure 5A). Interestingly, we observed a significant increase in the percentage of active caspase 3^+^ cells in the DN, DP and CD4^+^ SP subpopulations from TβRIII^−/−^ fetal thymi compared to wild type thymi (Figure 5A). In accordance, MFI for active caspase 3 also showed an increase in this apoptotic mediator in DP and DN subpopulations, although the latter did not reach statistical significance, suggesting that DP thymocytes are more susceptible to die in TβRIII^−/−^ fetal thymi (Figure 5A). In addition, annexin V staining confirmed the increased apoptosis of DP and CD4^+^ SP thymocytes in the absence of TβRIII (Figure 5B).

In agreement with our data, several findings highlight a crucial role for TβRIII as a regulator of apoptosis including the higher levels of caspase 3 and downregulation of the prosurvival factor Akt, which impact the integrity and function of liver [10]. In addition, overexpression of TβRIII leads to protection of cardiac fibroblasts from hypoxia-induced apoptosis by reversing caspase 3 activation, Bax upregulation and inducing Bcl-2 downregulation [36].

Although most of the functions mediated by TβRIII may involve the interaction with TGFβ members, we cannot rule out additional functions mediated by the intracellular domain of this co-receptor, as it has been recently involved in cell migration and apoptosis in a ligand independent fashion [37], [38]. Indeed, after shedding of the TβRIII ectodomain, the resulting transmembrane-cytoplasmic domain is cleaved by γ-secretase, influencing the TGFβ signaling response [39]. Thus, we may argue that this TβRIII cytoplasmic domain could trigger transduction signals in a similar fashion as Notch [40], acting as novel regulator of early T cell differentiation.

In summary, our data provide the first evidence that TβRIII plays a key role during T cell development: it is differentially expressed during T cell ontogeny and regulates DN-DP transition by protecting thymocytes from apoptosis. However, many questions remain unanswered concerning the role of TβRIII in T cell immunobiology. Indeed, since TGFβ is a critical regulator of T cell-mediated immunity, it is feasible to propose that the described effects of TGFβ members in immune cells may be regulated by TβRIII. In this sense, the generation of conditional knockout models for TβRIII will allow us to elucidate the function of this receptor in specific cell types, including mature T cell populations.

## Supporting Information

Figure S1 **TβRIII is expressed in lymphoid cells.** (A) Western blot assay of cell lysates obtained from thymus, spleen and lymph nodes, probed with monoclonal anti-TβRIII antibody. A band of a molecular weight of 100 kDa, corresponding to TβRIII core protein, was visualized in all samples tested. The bottom blot shows the corresponding loading control with anti-β-actin antibody, which detects a band of 42 kDa. (B) Analysis of specificity of TβRIII antiserum in TβRIII−/− fetal thymi. Upper panel, histograms show expression of TβRIII in gated DN and DP thymocytes from TβRIII+/+ and TβRIII−/− fetal thymi at day 3 of culture. Lower panel, representative histograms showing the detection of TβRIII in gated DN, DP, and SP thymocytes from TβRIII+/+ and TβRIII−/− fetal thymi at day 7 of culture. (C) Detection of surface TβRIII expression on thymocytes after competition with soluble form of TβRIII (TβRIIIs). Histograms show TβRIII staining in gated DN, DP and SP thymocytes in the presence of increasing doses of TβRIIIs.
(TIF)

Figure S2 **Analysis of thymocyte cellularity and DN immature subsets at days 3 and 7 of culture in the presence of TβRIII-blocking antibodies.** (A) Comparative graphs showing the numbers of total thymocytes obtained after 3 and 7 days of culture of E14 fetal thymic lobes, treated with anti-TβRIII or pre-immune antiserum (control lobe). (B) Left panel, representative CD44 versus CD25 staining dot plots are shown to compare the effects of both treatments. (C) Comparative graphs show the percentages of DN1, DN2, DN3, and DN4 immature thymocytes obtained at day 3 and 7 of culture. (D) Graphs show absolute cell numbers of DN immature subsets at day and 7 of culture, treated with anti-TβRIII or pre-immune antiserum. Data are representative of two independent experiments. Mean values ± SEM are shown (n  = 7 per group for day 3, and n  = 9 per group for day 7). Asterisks indicate statistically significant differences (* p≤0.05).
(TIF)

Figure S3 **Analysis of DN immature subsets in TβRIII−/− fetal thymi at days 3 and 7 of culture.** DN immature thymocytes from TβRIII+/+ and TβRIII−/− E14 fetal thymic lobes cultures were analyzed at day 3 and 7 of culture. (A) Left panel, representative CD44 versus CD25 staining dot plots are shown to compare the percentages between both genotypes. Right panel, comparative graphs show the percentages of DN1, DN2, DN3, and DN4 immature thymocytes obtained at day 3 and 7 of culture. (B) Graphs show absolute cell numbers of DN immature subsets from TβRIII+/+ and TβRIII−/− fetal thymi at day 3 and 7 after culture. Data are representative of three independent experiments. Mean values ± SEM are shown, TβRIII+/+ n  = 17 and TβRIII−/− n  = 16. Asterisks indicate statistically significant differences (* p≤0.05).
(TIF)
