# Supplementary figures and images for: Betaglycan (TβRIII) Is Expressed in the Thymus and Regulates T Cell Development by Protecting Thymocytes from Apoptosis
Source: PLoS One. 2012 Aug 29;7(8):e44217. doi: 10.1371/journal.pone.0044217 (PMC3430661; doi:10.1371/journal.pone.0044217)

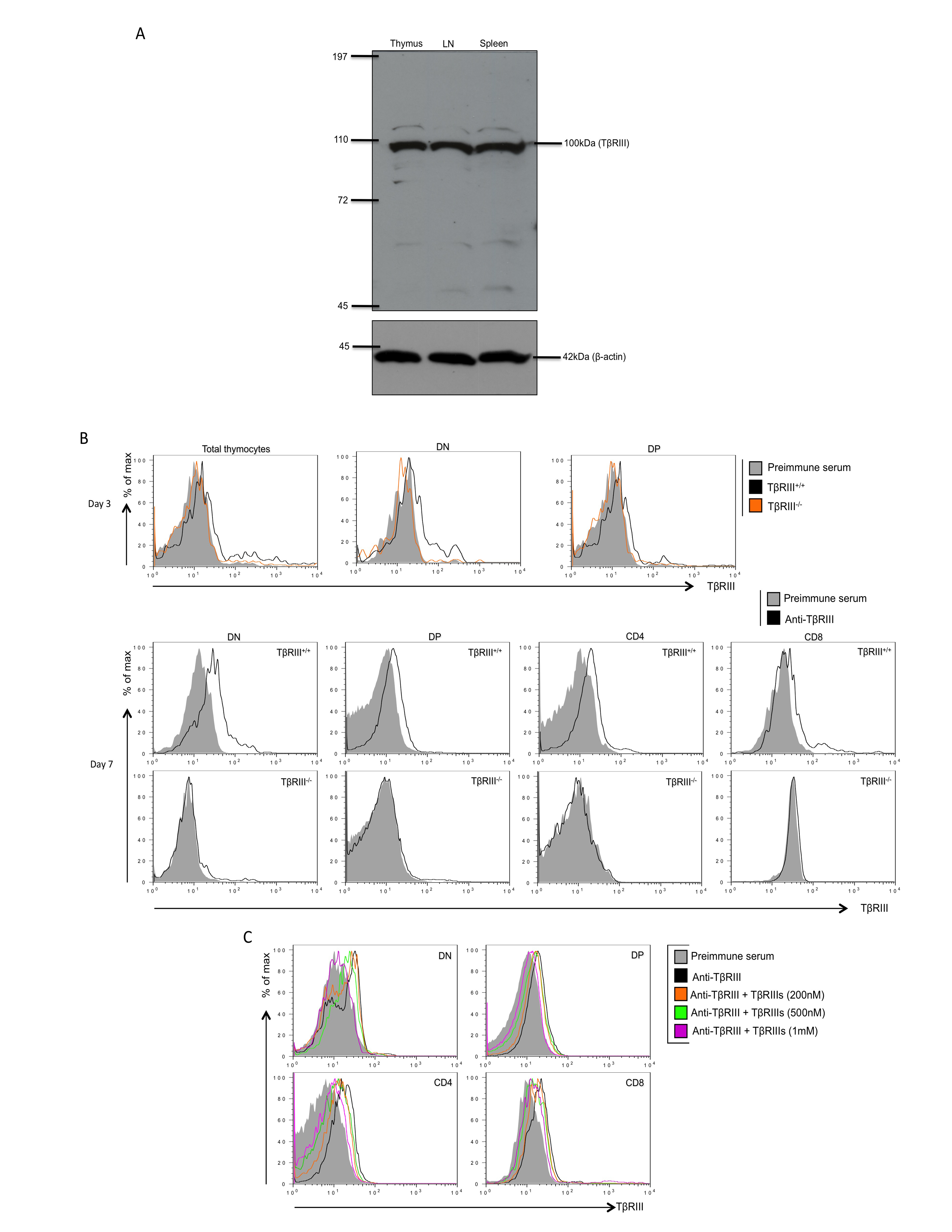

Supplement: Figure S1 — TβRIII is expressed in lymphoid cells. (A) Western blot assay of cell lysates obtained from thymus, spleen and lymph nodes, probed with monoclonal anti-TβRIII antibody. A band of a molecular weight of 100 kDa, corresponding to TβRIII core protein, was visualized in all samples tested. The bottom blot shows the corresponding loading control with anti-β-actin antibody, which detects a band of 42 kDa. (B) Analysis of specificity of TβRIII antiserum in TβRIII−/− fetal thymi. Upper panel, histograms show expression of TβRIII in gated DN and DP thymocytes from TβRIII+/+ and TβRIII−/− fetal thymi at day 3 of culture. Lower panel, representative histograms showing the detection of TβRIII in gated DN, DP, and SP thymocytes from TβRIII+/+ and TβRIII−/− fetal thymi at day 7 of culture. (C) Detection of surface TβRIII expression on thymocytes after competition with soluble form of TβRIII (TβRIIIs). Histograms show TβRIII staining in gated DN, DP and SP thymocytes in the presence of increasing doses of TβRIIIs. (TIF) [file pone.0044217.s001.tif]

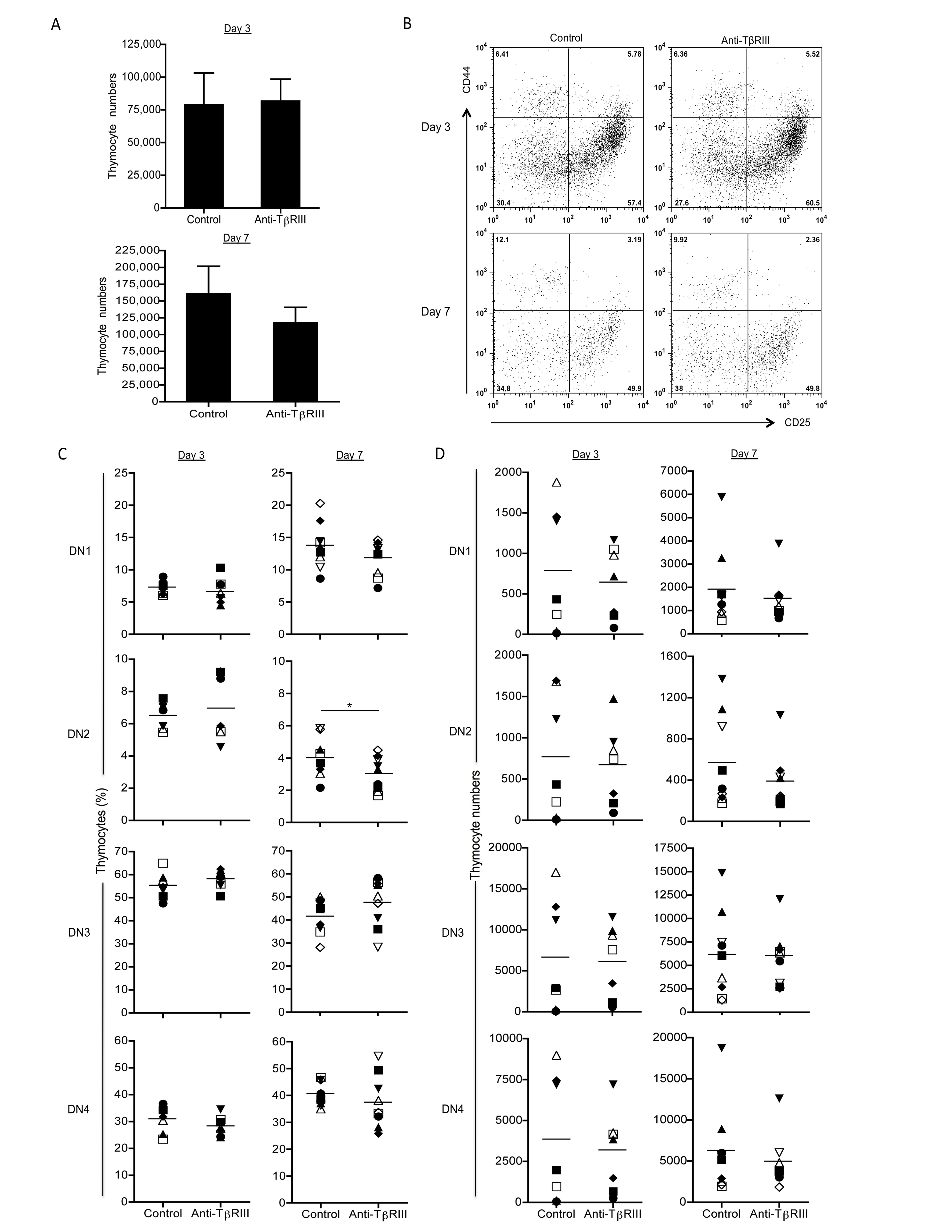

Supplement: Figure S2 — Analysis of thymocyte cellularity and DN immature subsets at days 3 and 7 of culture in the presence of TβRIII-blocking antibodies. (A) Comparative graphs showing the numbers of total thymocytes obtained after 3 and 7 days of culture of E14 fetal thymic lobes, treated with anti-TβRIII or pre-immune antiserum (control lobe). (B) Left panel, representative CD44 versus CD25 staining dot plots are shown to compare the effects of both treatments. (C) Comparative graphs show the percentages of DN1, DN2, DN3, and DN4 immature thymocytes obtained at day 3 and 7 of culture. (D) Graphs show absolute cell numbers of DN immature subsets at day and 7 of culture, treated with anti-TβRIII or pre-immune antiserum. Data are representative of two independent experiments. Mean values ± SEM are shown (n = 7 per group for day 3, and n = 9 per group for day 7). Asterisks indicate statistically significant differences (* p≤0.05). (TIF) [file pone.0044217.s002.tif]

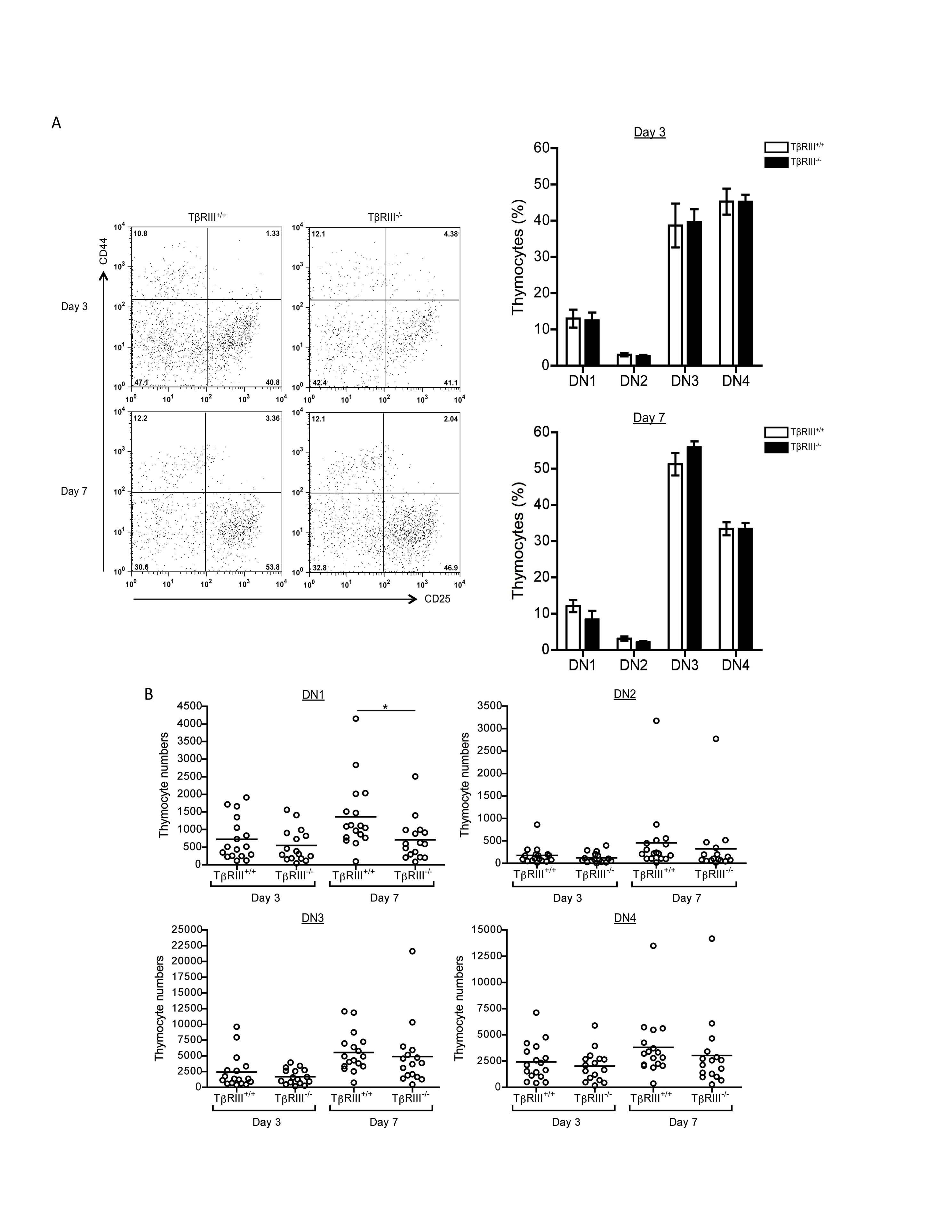

Supplement: Figure S3 — Analysis of DN immature subsets in TβRIII−/− fetal thymi at days 3 and 7 of culture. DN immature thymocytes from TβRIII+/+ and TβRIII−/− E14 fetal thymic lobes cultures were analyzed at day 3 and 7 of culture. (A) Left panel, representative CD44 versus CD25 staining dot plots are shown to compare the percentages between both genotypes. Right panel, comparative graphs show the percentages of DN1, DN2, DN3, and DN4 immature thymocytes obtained at day 3 and 7 of culture. (B) Graphs show absolute cell numbers of DN immature subsets from TβRIII+/+ and TβRIII−/− fetal thymi at day 3 and 7 after culture. Data are representative of three independent experiments. Mean values ± SEM are shown, TβRIII+/+ n = 17 and TβRIII−/− n = 16. Asterisks indicate statistically significant differences (* p≤0.05). (TIF) [file pone.0044217.s003.tif]
